# Ethylene Potentiates Sulfur-Mediated Reversal of Cadmium Inhibited Photosynthetic Responses in Mustard

**DOI:** 10.3389/fpls.2016.01628

**Published:** 2016-11-02

**Authors:** Nafees A. Khan, Mohd Asgher, Tasir S. Per, Asim Masood, Mehar Fatma, M. I. R. Khan

**Affiliations:** Plant Physiology and Biochemistry Laboratory, Department of Botany, Aligarh Muslim UniversityAligarh, India

**Keywords:** cadmium, ethylene, norbornadiene, photosynthesis, sulfur

## Abstract

The potential of exogenous ethylene and sulfur (S) in reversal of cadmium (Cd)-inhibited photosynthetic and growth responses in mustard (*Brassica juncea* L. cv. Pusa Jai Kisan) were studied. Plants grown with 50 μM Cd showed increased superoxide and H_2_O_2_ accumulation and lipid peroxidation together with increased activity of 1-aminocyclopropane carboxylic acid synthase (ACS) and ethylene production and inhibition of photosynthesis and growth. Application of 1 mM SO_4_^2-^ or 200 μL L^-1^ ethephon (ethylene source) influenced photosynthetic and growth performance equally in presence or absence of Cd. However, their combined application synergistically improved photosynthetic performance more in presence of Cd and reduced oxidative stress (lower superoxide and H_2_O_2_ accumulation) by decreasing ethylene and glucose sensitivity with the increase in cysteine and methionineand a non-proteinogenic thiol (reduced glutathione; GSH) contents. The central role of ethylene in potentiating S-mediated reversal of Cd-induced oxidative stress was evident with the use of ethylene action inhibitor, norbornadiene (NBD). The application of NBD resulted in decreased thiol production and photosynthetic responses. This suggests that ethylene promotes the effects of S in reversal of adverse effects of Cd, and thus, ethylene modulation may be considered as potential tool to substantiate the S effects in reversal of Cd inhibited photosynthesis and growth in mustard.

## Introduction

Cadmium (Cd) with long biological half-life and potentially toxic even at low concentration has become one of the most challenging threats due to its increasing concentration in agricultural system (Choppala et al., 2014; Asgher et al., 2015; Khan et al., 2015a; Wahid and Khaliq, 2015). Cadmium alters chloroplast structure, inhibits nutrients uptake, inactivates enzymes of CO_2_ fixation and negatively affects various cellular functions through displacement of essential elements from proteins (Mobin and Khan, 2007; DalCorso et al., 2008; Masood et al., 2012b; Choppala et al., 2014). It accumulates to high levels in leaves and adversely impacts chlorophyll (Chl) biosynthesis and photosynthetic process by disrupting the electron transport chain, and aggregation of pigment protein complexes of the photosystems (Baryla et al., 2001; Liu et al., 2011; Shukla et al., 2014). Excess of Cd reduces plant performance due to increased formation of reactive oxygen species (ROS) in plants. Plants develop an array of mechanisms including production of thiol compounds that are regarded as essential components for ROS homeostasis (Anjum et al., 2012; Asgher et al., 2014; Kovácik et al., 2014). The decrease in growth under Cd stress results from the inhibition of photosynthesis (Masood et al., 2012b; Asgher et al., 2014; Khan et al., 2015b). Recently, Liu et al. (2014) have reported that the decrease in photosynthesis with Cd stress was related to the inhibition of maximal photochemical efficiency, quantum yield of electron transport, photochemical quenching and electronic transport rate in *Gossypium hirsutum*. Several efforts have been made to counteract the Cd-induced toxicity and restore the photosynthetic capacity of plants. Supplementation of plants with mineral elements is one of the better strategies adopted. Sulfur (S) limitations resulted in the chlorosis of young leaves and inhibition of photosynthetic activity (Hawkesford, 2000), whereas S supply increases photosynthetic capacity of leaves through an increase in stromal and thylakoid protein and regulating stomatal movement under Cd stress (Masood et al., 2012b; Asgher et al., 2014; Khan et al., 2015b).

Sulfur is component of various metabolic compounds such as cysteine (Cys), methionine (Met), reduced glutathione (GSH), sulfolipids, iron-sulfur clusters, allyl Cys, and glucosinolates (Khan N.A. et al., 2014) that may be necessary for Cd tolerance (Anjum et al., 2008). It has been reported that Cys alleviates salt-induced growth inhibition and reduces oxidative stress by modulating cellular redox status due to its own antioxidant property in *Hordeum vulgare* plants (Genisel et al., 2014). Astolfi and Zuchi (2013) showed that adequate S allowed sufficient GSH synthesis that helped in avoiding the effects of ROS on photosynthetic efficiency and growth to cope with salt stress. The increased accumulation of proteinogenic and non-proteinogenic thiols with increasing Cd concentrations in plants suggested that efficient sulfate uptake and assimilation may help in Cd tolerance (Nocito et al., 2002; Sun et al., 2007; Lancilli et al., 2014). The S deficiency leads to reduced Chl content, pigment system (PS) II efficiency, and ribulose 1,5-bisphosphate carboxylase oxygenase (Rubisco) content (Lunde et al., 2008). Sulfur may also regulate photosynthesis through ethylene production as ethylene synthesis is linked to S-assimilation via the formation of Cys and Met. Thus, the S-mediated changes in growth and development of plants also involve ethylene. Further, photosynthetic protection under Cd stress through S-mediated ethylene may also involve glucose (Glc) as antagonistic interaction between Glc and ethylene exists (Yanagisawa et al., 2003). Iqbal et al. (2011) reported that ethephon and N together increased ethylene and decreased Glc sensitivity, which increased photosynthesis and growth.

Phytohormones play key role in the regulation of photosynthetic characteristics (Khan et al., 1998, 2002, 2005; Masood and Khan, 2013; Asgher et al., 2014; Iqbal et al., 2014). Ethylene is a gaseous plant hormone with versatile role in regulating photosynthesis and growth responses under abiotic stress (Wang et al., 2013; Khan and Khan, 2014; Schellingen et al., 2014; Asgher et al., 2015; Thao et al., 2015; Khan et al., 2015c). Fu et al. (2014) studied the regulatory interaction between S-assimilation and ethylene for tolerance to arsenic in *Arabidopsis* Col-0. Exogenously applied GSH positively modulates ethylene biosynthetic pathways enzyme and improves stress tolerance in *Arabidopsis* Col-0 showing that GSH mediated resistance to stresses occurs via an ethylene signaling pathway (Datta et al., 2015). Recent studies showed that ethylene signaling pathways might be involved in the accumulation of GSH under Cd stress (Guan et al., 2015). Recently, Iqbal et al. (2015) have shown that ethylene and N interact to regulate photosynthesis of salt stressed *Brassica juncea*. Nazar et al. (2014) have shown the involvement of ethylene in reversal of salt inhibited photosynthesis by S in *B. juncea*. Study of Masood et al. (2012b) has shown that ethylene alleviated Cd-induced photosynthetic inhibition by S in *B. juncea*. However, the interaction of ethylene with S and antioxidant metabolites to regulate efficiency of PSII and photosynthesis in *B. juncea* under Cd stress has not been worked out. The aim of the research was to elucidate the role of ethylene in modulating antioxidants in presence of S and alleviation of Cd stress in *B. juncea* plants.

## Materials and Methods

### Plants Material and Growth Conditions

Seeds of mustard (*Brassica juncea* L. Czern and Coss. cv. Pusa Jai Kisan) were selected for the study based on our earlier findings of Iqbal et al. (2012b), which showed that Pusa Jai Kisan was sensitive to ethylene. In order to assess the role of ethylene in S-mediated alleviation of Cd stress, seeds were grown individually with 0 or 50 μM Cd. In addition, plants were grown with 1.0 mM SO_4_^2-^, 200 μL L^-1^ ethephon, 1 mM SO_4_^2-^ plus 200 μL L^-1^ ethephon in presence or absence of Cd.

Healthy seeds were surface sterilized with 0.01% HgCl_2_ followed by repeated washings with distilled water. The seeds were sown in 23-cm diameter pots filled with acid-washed sand. Two healthy plants of nearly equal size in each pot were maintained and were fed with 250 mL of modified full strength Hoagland nutrient solution containing 3 mM KNO_3_, 2 mM Ca(NO_3_)_2_, 1 mM NH_4_H_3_PO_4_, 50 μM KCl, 25 μM H_3_BO_4_, 2 μM MnCl_2_, 20 μM ZnSO_4_, 0.5 μM CuSO_4_, 0.5 μM (NH_4_)_6_Mo_7_O_24_, and 20 μM Na_2_Fe-EDTA on alternate days. MgSO_4_ was used for obtaining 1.0 mM SO_4_^2-^ concentration and a uniform Mg^2+^ concentration in the treatments was maintained by the addition of MgCl_2._ Plants grown in nutrient solution served as control. Sulfur was given along with the nutrient solution at 15 days after sowing (DAS), and ethephon at 200 μL L^-1^ concentrations was sprayed on foliage as an average of 10-12 mL per plant at 15 DAS. Cadmium was applied as CdCl_2_ in the nutrient solution.

In another experiment, to substantiate the finding that ethylene has a role in eliciting S-mediated alleviation of Cd stress, plants were grown either with 0 or 50 μM Cd or in combined treatment of 50 μM Cd + 1.0 mM SO_4_^2-^ + 200 μL L^-1^ ethephon and 50 μM Cd + 1.0 mM SO_4_^2-^ + 200 μL L^-1^ ethephon + 100 μM NBD. Treatment of ethephon or NBD was given at 15 DAS.

The pots were kept in a greenhouse of the Botany Department, Aligarh Muslim University, Aligarh, India under natural day/night conditions of 22/14 ± 3°C, photosynthetically active radiation (PAR; 680 μmol m^-2^ s^-1^) and relative humidity of 62 ± 5%. Treatments in both the experiments were arranged in a factorial randomized block design. The number of replicates for each treatment was four (*n* = 4). At 30 DAS, determinations were made for oxidative stress, glucose content, ethylene production, and S-assimilation, photosynthesis and growth characteristics in both the experiments.

### Determination of Cd Content

Cadmium content was determined in root and leaf samples. Leaves were washed with distilled water, and the roots were washed with an ice-cold 5 mM CaCl_2_ solution for 10 min to displace extracellular Cd (Rauser, 1987). Leaf and root samples were dried for 48 h at 80°C, ground to fine powder and digested with concentrated HNO_3_/HClO_4_ (3:1, v:v). Cadmium concentration was determined by atomic absorption spectrophotometer (GBC, 932 plus; GBC Scientific Instruments, Braeside, Australia).

### Determination of H_2_O_2_ Content and Lipid Peroxidation

The content of H_2_O_2_ was determined following the method of Okuda et al. (1991). Fresh leaf tissues (500 mg) were ground in ice-cold 200 mM perchloric acid. After centrifugation at 1,200 *g* for 10 min, perchloric acid of the supernatant was neutralized with 4 M KOH. The insoluble potassium perchlorate was eliminated by centrifugation at 500 *g* for 3 min. In a final volume of 1.5 mL, 1 mL of the eluate, 400 μL of 12.5 mM 3-(dimethylamino) benzoic acid (DMAB) in 0.375 M phosphate buffer (pH 6.5), 80 μL of 3-methyl-2-benzothiazoline hydrazone (MBTH) and 20 μL of peroxidase (0.25 unit) were added. The reaction was started by the addition of peroxidase at 25°C and the increase in absorbance was recorded at 590 nm.

Lipid peroxidation in leaves was determined by estimating the content of TBARS as described by Dhindsa et al. (1981). Fresh leaf tissues (500 mg) were ground in 0.25% 2-thiobarbituric acid (TBA) in 10% trichloroacetic acid (TCA) using mortar and pestle. After heating at 95°C for 30 min, the mixture was rapidly cooled on ice bath and centrifuged at 10,000 *g* for 10 min. To 1 mL aliquot of the supernatant 4 mL 20% TCA containing 0.5% TBA was added. The absorbance of the supernatant was read at 532 nm and corrected for non-specific turbidity by subtracting the absorbance of the same at 600 nm. The content of TBARS was calculated using the extinction coefficient (155 mM^-1^ cm^-1^).

### Histochemical Detection of ROS

For assay of the accumulation of 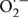 by histochemical staining method, nitro blue tetrazolium (NBT) was used to stain the leaves by adopting the method of Wang et al. (2011) with slight modification. The samples from each treatment were immersed into 1 mg/mL NBT solution prepared in 10 mM phosphate buffer (pH 7.8) at ambient temperature under light for 6 h. Blue (NBT staining) spots appeared; the stained samples were boiled in ethanol.

### Activities of ATP-Sulfurylase, SAT, and S Content

Activity of ATP-sulfurylase (ATP-S; EC, 2.7.7.4) was measured using molybdate-dependent formation of pyrophosphate as described by Lappartient and Touraine (1996). Fresh leaf tissue (1.0 g) was ground at 4°C in a buffer consisting of 10 mM Na_2_EDTA, 20 mM Tris-HCl pH 8.0, 2 mM dithiothreitol (DTT), and 0.01 g mL^-1^ polyvinyl pyrrolidone (PVP), using 1:4 (w/v) tissue to buffer ratio. The homogenate was centrifuged at 20,000 *g* for 10 min at 4°C. The supernatant was used for *in vitro* ATP-sulfurylase assay. The reaction was initiated by adding 0.1 mL of extract to 0.5 mL of the reaction mixture, which contained 7 mM MgCl_2_, 5 mM Na_2_MoO_4_, 2 mM Na_2_ATP, and 0.032 units mL^-1^ of sulfate-free inorganic pyrophosphate in 80 mM Tris–HCl buffer, pH 8.0. Another aliquot from the same extract was added to the same reaction mixture but without Na_2_MoO_4_. Incubations were carried out at 37°C for 15 min, after which phosphate was determined.

Activity of serine acetyltransferase (SAT; EC, 2.3.1.30) in the leaf extract was determined by the method of Kredich and Tomkins (1966). Fresh leaf tissue (0.5 g) was ground with a chilled mortar and pestle in 2 mL of ice cold extraction buffer 100 mM Tris–HCl pH 8.0, 100 mM KCl, 20 mM MgCl_2_, 1% Tween 80, and 10 mM DTT. The samples were centrifuged at 11,600 *g* for 10 min at 4°C. The supernatant obtained was used for SAT assay. The enzyme reaction mixture contained 0.1 mM acetyl CoA, 50 mM Tris–HCl pH 7.6, 1 mM DTNB, 1 mM EDTA, and 1 mM L-serine in 1 mL. Subsequent to reaction initiation by addition of enzyme at 25°C, the initial velocity was estimated by monitoring the increase in absorbance at 412 nm and the rates were calculated using an extinction coefficient of 13,600 for thionitrobenzoic acid. A blank containing all materials except L-serine was run simultaneously and subtracted from the reaction rate obtained with L-serine.

Sulfur content was determined in leaf samples digested in a mixture of concentrated HNO_3_ and 60% HClO_4_ (85:1 v/v) using the turbidimetric method (Chesnin and Yien, 1950).

### Determination of Cysteine, Methionine, Reduced Glutathione, and Redox State

Cysteine content in leaves was determined spectrophotometri-cally adopting the method of Giatonde (1967). Fresh leaf (500 mg) was homogenized in 5% (w:v) ice-cold perchloric acid. The suspension was centrifuged at 2,800 *g* for 1 h at 5°C and supernatant was filtered. After that, 1 mL of filtrate was treated with acid ninhydrin reagent and the absorption was read at 580 nm. The amount of Cys was calculated using the calibration curve obtained for standard Cys.

Methionine content was determined by the method of Horn et al. (1946). Fresh leaf sample (500 mg) was reflexed with 20.0 mL 6.0 N HCl for 20-24 h followed by evaporation on water bath with the addition of 1.0 g activated charcoal. The filtrate was collected to which 4.0 mL de-ionized water and 2.0 mL of 5 N-NaOH were added followed by the addition of 0.1 mL sodium nitropruside and 2.0 mL glycine solution (3%). Finally, 4.0 ml phosphoric acid was added and color intensity was read at 450 nm.

Reduced glutathione content was determined following the method of Anderson (1985). Reduced glutathione was assayed by an enzymic recycling procedure in which it was sequentially oxidized by 5′5′-dithiobis-2-nitrobenzoic acid (DTNB) and reduced by NADPH in the presence of GR. For specific assay of GSSG, the GSH was masked by derivatization with 2-vinylpyridine. Fresh leaves (500 mg) were homogenized in 2.0 mL of 5% sulfosalicylic acid under cold conditions. The homogenate was centrifuged at 10,000 *g* for 10 min. To 0.5 mL of supernatant, 0.6 mL of phosphate buffer (100 mM, pH 7.0) and 40 μL of DTNB were added. After 2 min the absorbance was read at 412 nm.

Redox state was presented as the ratio of GSH to oxidized glutathione (GSSG).

### Determination of Glucose Content

Leaf glucose content was determined with the method of Krishnaveni et al. (1984) by using glucose as the standard. Leaf extract was prepared by extracting dried leaf in 80% ethanol. The extract was heated in a water bath at 60°C for 10 min and then cooled. The samples were then centrifuged at 1500 *g* for 1 min. The supernatant was used for Glc determination. The reaction mixture consisted of 25 mg *O*-dianisidine, 1 mL methanol, 49 mL of 0.1 M phosphate buffer pH 6.5, 5 mg of peroxidase, and 5 mg of glucose oxidase. The reaction was started by adding 0.5 mL of the extract to 1 mL of the reaction mixture in a test tube. The tubes were incubated at 35°C for 40 min and the reaction was terminated by addition of 2 mL of 6 N HCl. The color intensity was read at 540 nm.

### Measurement of ACS Activity and Ethylene Evolution

Activity of 1-aminocyclopropane carboxylic acid synthase (ACS; EC, 4.4.1.14) was measured by adopting the methods of Avni et al. (1994) and Woeste et al. (1999). Leaf tissue (5.0 g) was ground in 100 mM HEPES buffer (pH 8.0) containing 4 mM DTT, 2.5 mM pyridoxal phosphate, and 25% PVP. The homogenized preparation was centrifuged at 12,000 *g* for 15 min. One ml of the supernatant was placed in a 30 mL tube and 0.1 mL of 5 mM *S*-adenosyl methionine (AdoMet) was added and incubated for 2 h at 22°C. The ACC formed was determined by its conversion to ethylene by the addition of 0.1 mL of 20 mM HgCl_2_ followed by the addition of 0.1 mL of a 1:1 mixture of saturated NaOH/NaCl and placed on ice for 10 min. In the control set, AdoMet was not added.

Ethylene was measured by cutting 0.5 g of leaf material into small pieces that were placed into 30 mL tubes containing moist paper to minimize evaporation from the tissue and were stoppered with secure rubber caps and placed in light for 2 h under the same condition used for plant growth. An earlier experiment showed that 2 h incubation time was adequate for ethylene detection without the interference of wound-induced ethylene, which began after 2 h of leaf incubation. A 1 mL gas sample from the tubes was withdrawn with a hypodermic syringe and assayed on a gas chromatograph (Nucon 5700, New Delhi, India) equipped with a 1.8 m Porapack N (80–100 mesh) column, a flame ionization detector and data station. Nitrogen was used as the carrier gas. The flow rates of nitrogen, hydrogen and oxygen were 30, 30, and 300 mL min^-1^, respectively. The detector was set at 150°C. Ethylene was identified based on the retention time and quantified by comparison with peaks from standard ethylene concentration.

### Determination of Gas Exchange Parameters, Rubisco Activity, and Growth Characteristics

Net photosynthetic rate, stomatal conductance, and intercellular CO_2_ concentration were measured in fully expanded uppermost intact leaves of plants in each treatment using infrared gas analyzer (CID-340, Photosynthesis System, Bio-Science, USA). The measurements were done between 11.00 and 12.00 h at light saturating intensity (PAR; 720 μmol m^-2^ s^-1^) and at 370 ± 75 μmol mol^-1^ atmospheric CO_2_ concentrations.

The activity of Rubisco (EC; 4.1.1.39) was determined spectrophotometrically by adopting the method of Usuda (1985) by monitoring NADH oxidation at 30^o^C at 340 nm. For enzyme extraction, leaf tissue (1.0 g) was homogenized using a chilled mortar and pestle with ice-cold extraction buffer containing 0.25 M Tris–HCl (pH 7.8), 0.05 M MgCl_2_, 0.0025 M EDTA, and 37.5 mg DTT. The homogenate was centrifuged at 4°C at 10,000 *g* for 10 min. The resulting supernatant was used to assay the enzyme. The reaction mixture (3 mL) contained 100 mM Tris-HCl pH 8.0, 40 mM NaHCO_3_, 10 mM MgCl_2_, 0.2 mM NADH, 4 mM ATP, 5 mM DTT, 1U of glyceraldehyde 3-phosphodehydrogenase, and 1U of 3-phosphoglycerate kinase and 0.2 mM ribulose 1,5-bisphosphate (RuBP). Protein was estimated according to Bradford (1976) using bovine serum albumin as standard.

Plants were uprooted carefully from the pots, washed to remove dust. Leaf area was measured with a leaf area meter (LA 211, Systronics, New Delhi, India). Dry mass of plants was recorded after drying the sample in a hot air oven at 80°C till constant weight.

### Measurement of Chlorophyll Fluorescence Parameters

Fully expanded leaves were allowed to adapt under dark condition for 30 min before chlorophyll fluorescence measurements using Junior-PAM chlorophyll fluorometer (Heinz Walz, Germany). Minimal fluorescence (*F_o_*) and maximum fluorescence (*F_m_*) were measured in dark-adapted leaves with a low measuring beam at a light intensity of 125 μmol m^-2^ s^-1^, whereas under light-adapted condition, minimal fluorescence (*F_o_*′) and maximum fluorescence (*F_m_*′) were measured in the same leaves with a saturating light intensity (720 μmol m^-2^s^-1^) together with steady-state fluorescence (*F_s_*). The variable fluorescence (*F_v_* and *F_v_*′) was calculated using the values of *F_m_*–*F_o_* and *F_m_*′–*F_o_*′, and Φ PS II was determined as *F_m_*′_-_*F*_s_/*F*_m_′, maximal efficiency of PS II by using *F_v_*/*F_m_* and intrinsic efficiency of PS II by using *F_v_*′/*F_m_*′. Using fluorescence parameters determined in both light- and dark-adapted states, the photochemical quenching (qP) and non-photochemical quenching (NPQ) were calculated. qP was calculated as (*F_m_*′–*F_s_*)/*F_v_*′ and NPQ as (*F_m_*–*F_m_*′)/*F_m_*′ (Maxwell and Johnson, 2000). Electron transport rate (ETR) was calculated by following formula: Φ PS II × photosynthetic photon flux density × 0.5 × 0.84 as suggested by Krall and Edwards (1992).

### Statistical Analysis

Data were analyzed statistically and standard errors were calculated. Analysis of variance was performed on the data using SPSS (ver. 17.0 Inc., USA) to determine the significance at *P* < 0.05. Least significant difference (LSD) was calculated for the significant data to identify difference in the mean of the treatment. Data are presented as mean ± SE (*n* = 4).

## Results

### Influence of Ethylene or/and S on Cd Accumulation

**FIGURE 1:**
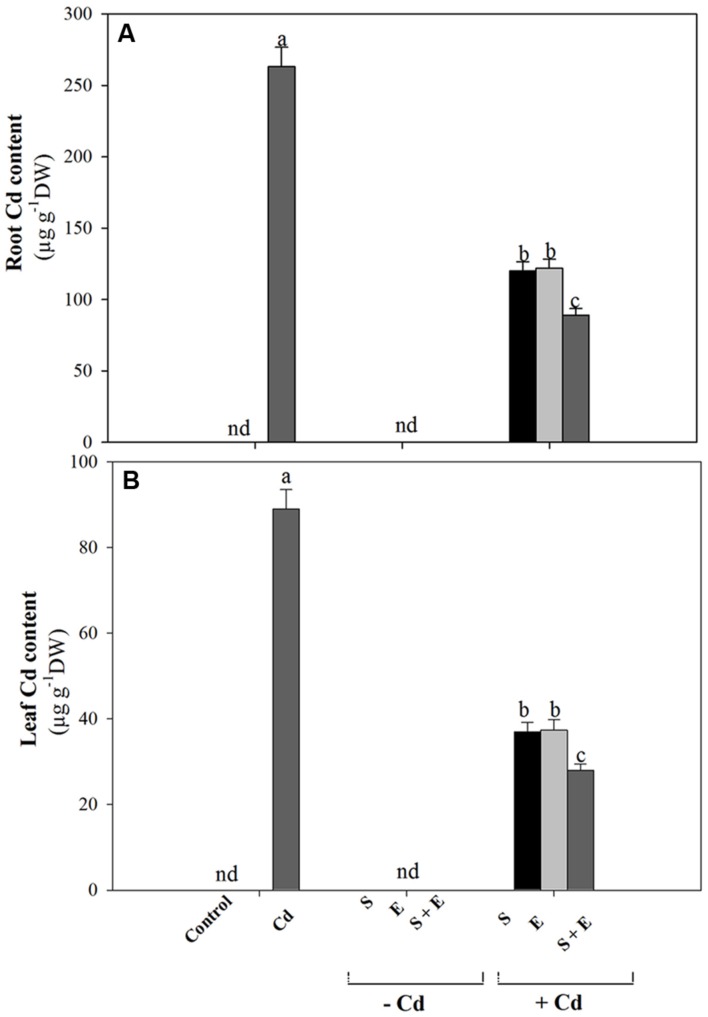
**Accmulation of Cd in root **(A)** and leaf **(B)** of mustard (*Brassica juncea* L.) at 30 DAS.** Plants were grown individually with 0 or 50 μM Cd or with 1 mM SO_4_^2-^(S), 200 μL L^-1^ ethephon (E), 1 mM SO_4_^2-^ plus 200 μL L^-1^ ethephon in presence or absence of Cd. Data are presented as treatments mean ± SE (*n* = 4). Data followed by same letter are not significantly different by LSD test at *P* < 0.05. nd, not determined.

Cadmium treatment resulted in higher root and leaf Cd content than control. Application of either S or ethephon reduced root Cd content equally by about 50% compared to Cd treated plants. The combination of S + ethephon maximally reduced Cd content by about 66% in root. In leaf, the S or ethephon applied individually reduced Cd content equally by about 58%, and the combined S plus ethephon reduced Cd content by about 68% compared to the Cd treated plants (**Figures 1A,B**).

### Influence of Ethylene or/and S under Cd Stress

#### Oxidative Stress

The samples originated from Cd-treated plants showed higher values for content of TBARS and H_2_O_2_ by 2.7-times and 2.3-times, respectively, compared to control. The observed values for these characteristics in plants receiving S or ethephon without Cd showed that S or ethephon reduced TBARS and H_2_O_2_ content equally compared to control. However, the combined treatment of S and ethephon more prominently reduced oxidative stress. The application of S plus ethephon reduced TBARS content by 3.7-times and H_2_O_2_ content by 2.7-times compared to control (**Table 1**).

**Table 1:** Content of TBARS (nmol g^-1^ FW) and H_2_O_2_ (nmol g^-1^ FW), activity of ATP-S (U mg^-1^protein min^-1^), SAT (U mg^-1^protein min^-1^), and S content (mg g^-1^ DW) in mustard (*Brassica juncea* L.) at 30 DAS.

Parameters	Control	Cd	*S*	*E*	S + E	*Cd* + *S*	*Cd* + *E*	*Cd* + *S* + *E*
TBARS content	7.4 ± 0.48d	19.8 ± 1.30a	3.2 ± 0.21e	3.0 ± 0.19e	2.0 ± 0.13f	13.0 ± 0.85b	13.2 ± 0.86b	10.1 ± 0.66c
H_2_O_2_ content	49.3 ± 2.72d	114.4 ± 6.32a	30.1 ± 1.66e	29.8 ± 1. 64e	18.2 ± 1.00f	65.8 ± 3.63b	65.6 ± 3.62b	54.4 ± 3.00c
ATP-S activity	1.70 ± 0.07f	2.42 ± 0.10e	2.93 ± 0.12d	2.97 ± 0.12d	3.94 ± 0.17b	3.58 ± 0.15c	3.62 ± 0.15c	4.60 ± 0.20a
SAT activity	1.82 ± 0.24f	2.43 ± 0.13e	2.98 ± 0.16d	3.0 ± 0.15d	4.0 ± 0.21b	3.51 ± 0.18c	3.62 ± 0.19c	4.71 ± 0.25a
Sulfur content	4.8 ± 0.15e	3.5 ± 0.12f	6.8 ± 0.29b	6.8 ± 0.30b	7.5 ± 0.31a	5.7 ± 0.23d	5.7 ± 0.25d	6.3 ± 0.26c


*Plants were grown individually with 0 or 50 μM Cd or with 1 mM SO_4_^2-^ (S), 200 μL L^-1^ ethephon (E), 1 mM SO_4_^2-^ plus 200 μL L^-1^ ethephon in presence, or absence of Cd. Data are presented as treatments mean ± SE (*n* = 4). Data followed by same letter are not significantly different by LSD test at *P*** <** 0.05.*

### ROS Accumulation

**FIGURE 2:**
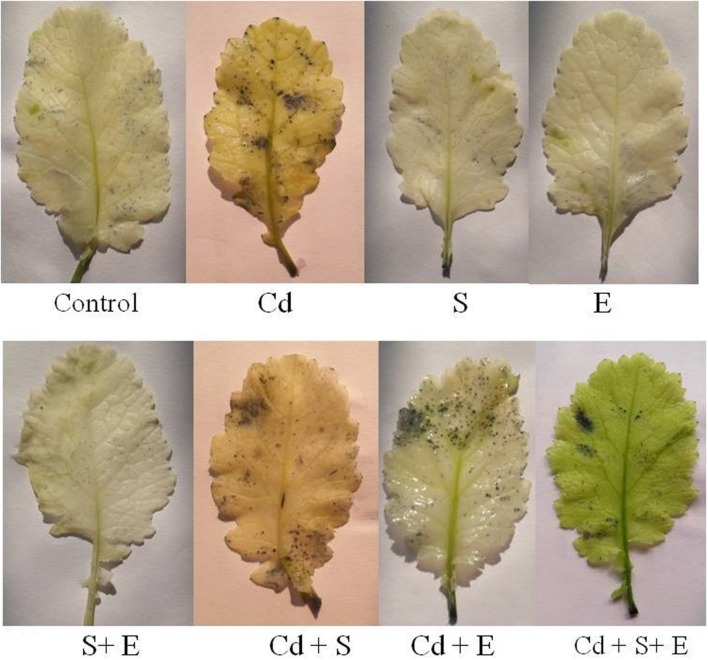
**Accmulation of superoxide ion by NBT staining in mustard (*Brassica juncea* L.) at 30 DAS.** Plants were grown individually with 0 or 50 μM Cd or with 1 mM SO_4_^2-^ (S), 200 μL L^-1^ ethephon (E), 1 mM SO_4_^2-^ plus 200 μL L^-1^ ethephon in presence or absence of Cd.

In Cd treated plants increase in 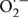 was observed as scattered dark blue spots in the leaf compared with the control (**Figure 2**). However, plants grown with combined treatment of S and ethephon more prominently diminished 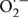 accumulation than the individual treatment of S and ethephon compared to control in absence of Cd. On the other hand, applying combined dose of S plus ethephon more prominently reduced the accumulation of 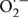 than individual application of S and ethephon in the presence of Cd compared to Cd-treated plants (**Figure 2**).

### Activity of ATP-S, SAT, and Content of S

Activity of ATP-S and SAT increased with Cd treatment by 42.3 and 33.5%, respectively, whereas S content decreased by 27.0% in comparison to control. Application of S or ethephon equally increased ATP-S activity by about 72.3%, SAT activity by 63.6% and S content by 41.6% over the control under non-stress condition. The combined treatment of S and ethephon enhanced ATP-S activity by 2.3-times, SAT activity by 2-times and S content by 56.2% compared to control. In the presence of Cd, application of S and ethephon together increased ATP-S activity by 2.7-times and SAT activity by 2.6-times and S content by 31.2% compared to control (**Table 1**).

### Content of Cys, Met, GSH, and Redox State

Sulfur assimilation and ethylene formation are linked through Cys formation. Cysteine is a common metabolite for the formation of thiols and ethylene. Sulfur availability and its assimilation control thiols synthesis which potentially protects plants against stress.

**FIGURE 3:**
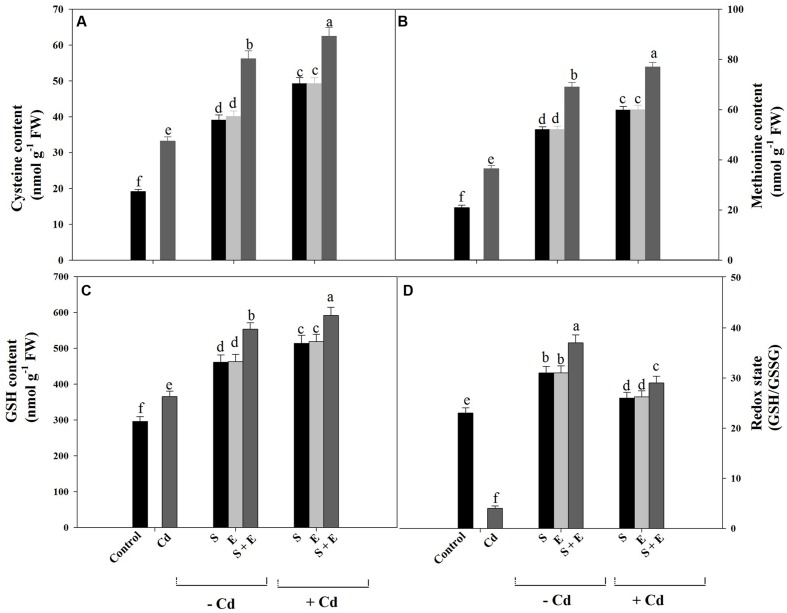
**Content of Cys **(A)**, Met **(B)**, GSH **(C)**, and redox state **(D)** in mustard (*Brassica juncea* L.) at 30 DAS.** Plants were grown individually with 0 or 50 μM Cd or with 1 mM SO_4_^2-^ (S), 200 μL L^-1^ ethephon (E), 1 mM SO_4_^2-^ plus 200 μL L^-1^ ethephon in presence or absence of Cd. Data are presented as treatments mean ± SE (*n* = 4). Data followed by same letter are not significantly different by LSD test at *P* < 0.05.

Cadmium stress significantly increased content of Cys and Met compared to control. Sulfur or ethephon equally increased Cys content by 2-times and Met content by 2.5-times, whereas combination of S and ethephon proved effective in increasing Cys content by 2.9-times and Met content by 3.2-times compared to control. The combination of S and ethephon in the presence of Cd maximally increased Cys content by 3.2-times and Met content by 3.6-times compared to control (**Figures 3A,B**).

Cadmium stress significantly increased GSH content by 23.3% compared to control. Sulfur or ethephon increased GSH content equally by 1.5-times whereas combination of S and ethephon proved effective in increasing GSH content by about 2-times in the absence of Cd compared to control. In the presence of Cd, S plus ethephon reversed the effect of Cd and increased GSH content by 2.3-times compared to control (**Figure 3C**).

The ratio of GSH to GSSG was used to indicate the redox state of plants under different treatments. Redox state decreased under Cd stress by 6-times compared to control. The application of individual S or ethephon proved equally effective in increasing redox state by 30.4% compared to control. The combination of S and ethephon significantly and maximally increased redox state by 54.1% in plants grown without Cd compared to control. The combination of S and ethephon in the presence of Cd maximally increased redox state by 26.0% compared to control (**Figure 3D**).

### Glucose Content

**FIGURE 4:**
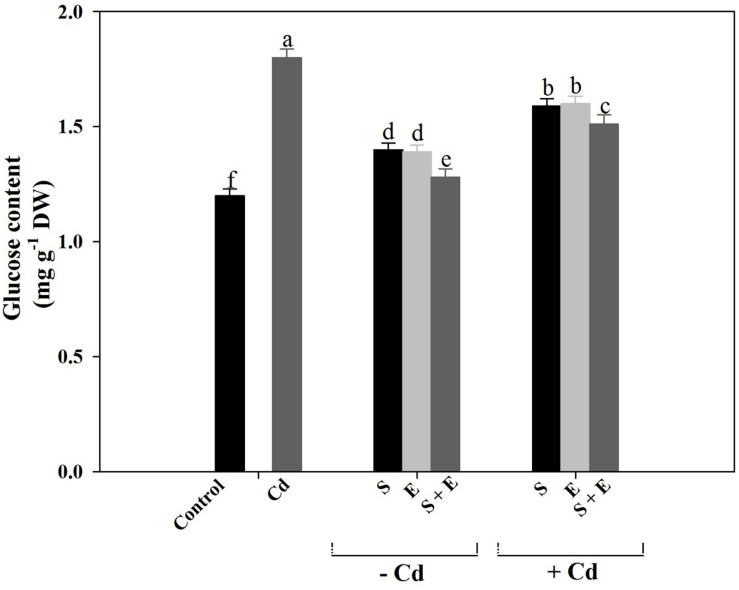
**Glucose content in mustard (*Brassica juncea* L.) at 30 DAS.** Plants were grown individually with 0 or 50 μM Cd or with 1 mM SO_4_^2-^ (S), 200 μL L^-1^ ethephon (E), 1 mM SO_4_^2-^ plus 200 μL L^-1^ ethephon in presence or absence of Cd. Data are presented as treatments mean ± SE (*n* = 4). Data followed by same letter are not significantly different by LSD test at *P* < 0.05.

Cadmium stress increased glucose content by 50.0% compared to control. Application of S or ethephon increased glucose content equally by 16.2%. Plant receiving S or ethephon in the presence of Cd showed a reduction of glucose content by 11.3% compared to Cd-treated plants. Application of S plus ethephon in the presence of Cd showed a maximum reduction in glucose content by 16.1% compared to Cd-treated plants (**Figure 4**).

### ACS Activity and Ethylene Production

**FIGURE 5:**
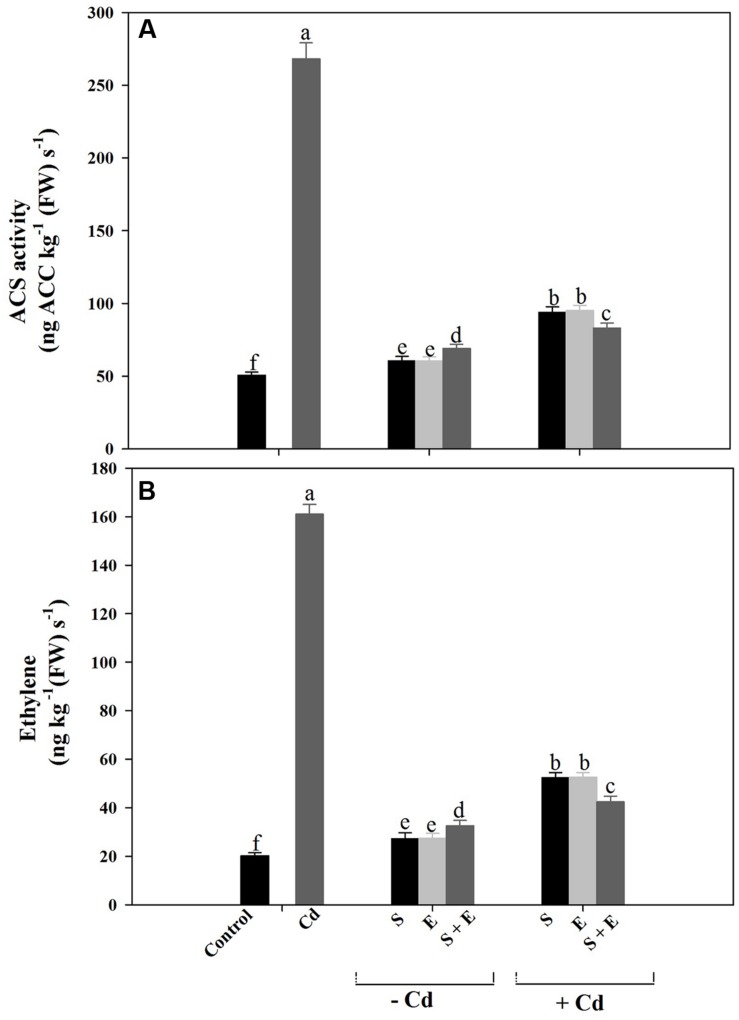
**1-aminocyclopropane carboxylic acid synthase (ACS) activity **(A)** and ethylene production **(B)** in mustard (*Brassica juncea* L.) at 30 DAS.** Plants were grown individually with 0 or 50 μM Cd or with 1 mM SO_4_^2-^ (S), 200 μL L^-1^ ethephon (E), 1 mM SO_4_^2-^ plus 200 μL L^-1^ ethephon in presence or absence of Cd. Data are presented as treatments mean ± SE (*n* = 4). Data followed by same letter are not significantly different by LSD test at *P* < 0.05.

Plants grown with Cd showed increased ACS activity and ethylene production compared to control plants. Cadmium stress increased ACS activity by 5.2-times and ethylene production by 8-times compared to control. Although individual or combined treatment of S and ethephon also increased ACS activity and ethylene production, however, the increase was lesser than Cd treatment. Plants receiving S or ethephon in the presence of Cd equally reduced ACS activity and ethylene production by 3-times compared to Cd-treated plants. The combination of S and ethephon in the presence of Cd maximally reduced ACS activity and ethylene production by 3.5-times compared to Cd-treated plants (**Figures 5A,B**).

### Photosynthetic Characteristics

Cadmium stress reduced gas exchange parameters, chlorophyll content and Rubisco activity compared to control. Plants grown in the absence of Cd showed higher photosynthesis with S or ethephon compared to control. In the presence of Cd, plants receiving S and ethephon completely alleviated Cd stress and promoted photosynthesis compared to control. Plants receiving S or ethephon equally increased net photosynthesis by 49.2%, stomatal conductance by 21.9%, intercellular CO_2_ concentration by 21.4%, chlorophyll content by 36.9% and Rubisco activity by 50.6% in comparison to control in the absence of Cd. Adding S and ethephon together increased net photosynthesis by 73.0%, stomatal conductance by 33.2%, intercellular CO_2_ concentration by 33.7%, Chl content by 53.1% and Rubisco activity by 74.6%, compared to control in plants not receiving Cd. Plants treated with Cd and received S or ethephon proved equally effective in alleviating the Cd-induced oxidative stress and enhanced net photosynthesis by 28.6%, stomatal conductance by 6.3%, intercellular CO_2_ concentration by 7.1%, Chl content by 12.9%, and Rubisco activity by 20.4%, compared to control. The combined treatment of S and ethephon maximally alleviated Cd stress and improved net photosynthesis by 42.3%, stomatal conductance by 19.3%, intercellular CO_2_ concentration by 19.4%, Chl content by 22.3%, and Rubisco activity by 37.4%, compared to control (**Table 2**).

**Table 2:** Net photosynthesis (μmol CO_2_ m^-2^s^-1^), stomatal conductance (mmol CO_2_ m^-2^ s^-1^), intercellular CO_2_ concentration (μmol CO_2_ mol^-1^), Rubisco activity (μmol CO_2_ mg^-1^ protein min^-1^), chlorophyll content (SPAD value), Actual PS II efficiency, Maximum PS II efficiency, Intrinsic PS II efficiency, Photochemical quenching (qP), Non-Photochemical quenching (NPQ), Electron transport rate, leaf area (cm^2^ plant^-1^), and plant dry mass (g plant^-1^) in mustard (*Brassica juncea* L.) at 30 DAS.

Parameters	Control	Cd	*S*	*E*	*S + E*	Cd + S	Cd + E	Cd + S + E
Net photosynthesis	16.7 ± 0.83e	10.3 ± 0.50f	24.9 ± 1.20b	24.8 ± 1.20b	28.9 ± 1.4a	21.9 ± 1.06d	21.8 ± 1.05d	23.7 ± 1.15c
Stomatal conductance	324.4 ± 14.8e	294.3 ± 13.4f	411.6 ± 21.6b	412.2 ± 21.6b	432.2 ± 22.7a	352.1 ± 15.4d	353.4 ± 15.4d	386.8 ± 16.2c
Intercellular CO_2_ concentration	224.1 ± 10.4e	160.5 ± 7.3f	280.2 ± 13.0b	281.5 ± 13.0b	300.2 ± 13.9a	247.2 ± 11.4d	247.5 ± 11.5d	267.6 ± 12.4c
Rubisco activity	0.83 ± 0.03e	0.50 ± 0.02f	1.25 ± 0.061b	1.26 ± 0.062b	1.45 ± 0.070a	0.98 ± 0.042d	0.99 ± 0.046d	1.14 ± 0.051c
Chlorophyll content	32.2 ± 1.61e	21.3 ± 1.06f	44.1 ± 2.22b	44.4 ± 2.22b	49.3 ± 2.47a	36.4 ± 1.82d	36.4 ± 1.82d	39.4 ± 1.97c
Actual PS II efficiency	0.59 ± 0.026e	0.49 ± 0.022f	0.71 ± 0.032b	0.72 ± 0.032b	0.84 ± 0.035a	0.64 ± 0.028d	0.65 ± 0.028d	0.68 ± 0.029c
Maximum PS II efficiency	0.79 ± 0.017c	0.68 ± 0.016f	0.88 ± 0.020b	0.89 ± 0.020b	0.94 ± 0.023a	0.82 ± 0.019d	0.83 ± 0.019e	0.86 ± 0.022c
Intrinsic PS II efficiency	0.70 ± 0.020e	0.63 ± 0.019f	0.81 ± 0.025b	0.82 ± 0.026b	0.86 ± 0.029a	0.74 ± 0.021d	0.75 ± 0.022d	0.78 ± 0.023c
qP	0.83 ± 0.027c	0.69 ± 0.024f	0.92 ± 0.030b	0.93 ± 0.030b	0.97 ± 0.032a	0.86 ± 0.029d	0.86 ± 0.028d	0.89 ± 0.029e
NPQ	0.67 ± 0.031d	0.84 ± 0.044a	0.62 ± 0.030e	0.61 ± 0.030e	0.54 ± 0.028e	0.74 ± 0.035b	0.73 ± 0.035b	0.70 ± 0.033c
Electron transport rate	178.4 ± 8.7e	148.1 ± 7.2f	214.7 ± 10.5b	217.7 ± 10.7b	254.1 ± 12.5a	193.5 ± 9.5d	196.5 ± 9.6d	205.6 ± 10.1c
Leaf area	142.3 ± 5.95e	86.5 ± 3.61f	180.5 ± 7.55b	181.7 ± 7.60b	201.1 ± 8.40a	165.6 ± 6.92d	166.6 ± 6.97d	176.2 ± 7.37c
Plant dry mass	8.1 ± 0.54e	4.6 ± 0.30f	10.3 ± 0.68b	10.4 ± 0.69b	12.0 ± 0.80a	9.6 ± 0.64d	9.7 ± 0.64d	10.9 ± 0.72c


*Plants were grown individually with 0 or 50 μM Cd or with 1 mM SO_4_^2-^ (S), 200 μL L^-1^ ethephon (E), 1 mM SO_4_^2-^ plus 200 μL L^-1^ ethephon in presence or absence of Cd. Data are presented as treatments mean ± SE (*n* = 4). Data followed by same letter are not significantly different by LSD test at *P****<*** 0.05.*

### PS II Activity

Cadmium stress reduced PS II activity [actual PS II efficiency, maximum PS II efficiency, intrinsic PS II efficiency, and qP and electron transport rate], however NPQ increased. Cadmium stress decreased PS II efficiency (actual PS II efficiency by 16.9%, maximum PS II efficiency by 13.9% and intrinsic PS II efficiency by 10.0%, qP by 16.8% and electron transport rate by 16.9%) compared to control. However, NPQ increased with Cd stress by 25.3% compared to control. Sulfur or ethephon application to plants alone improved the above characteristics equally compared to control. Follow up treatment of S plus ethephon to Cd-treated plants proved more effective in improving actual PS II efficiency along with maximum PS II efficiency, intrinsic PS II efficiency, qP, and electron transport rate than Cd plus S and Cd plus ethephon plants compared to Cd stress. Sulfur or ethephon decreased NPQ by 9.0%, however S and ethephon together decreased NPQ by 18.1% compared to control. Plants receiving S or ethephon in presence of Cd equally increased NPQ more prominently than combined treatment of S and ethephon in the presence of Cd compared to control (**Table 2**).

### Growth Characteristics

Leaf area, and plant dry mass were inhibited by Cd stress, while S and ethephon increased leaf area by 26.8 and 27.6% and plant dry mass by 35.8 and 35.9% in the absence of Cd compared to control. Application of S + ethephon exhibited maximum leaf area with an increase of 41.3% and plant dry mass by 48.3% compared to control in the absence of Cd. Leaf area and plant dry mass increased with S or ethephon of Cd-treated plants, but the maximum increase was noted with combined treatments of S + ethephon (**Table 2**).

### Ethylene is Involved in S-mediated Alleviation of Cd Stress: Effects of Ethylene Action Inhibitor

From the results explained above it is evident that S and ethylene coordinately improved photosynthetic performance of plants under Cd stress by regulating S-assimilation and thiols production. To substantiate the findings that ethylene has a role in S-mediated alleviation of Cd stress, the ethylene action inhibitor, NBD was used and S-assimilation, ethylene production, glucose, photosynthetic and growth characteristics were studied.

**FIGURE 6:**
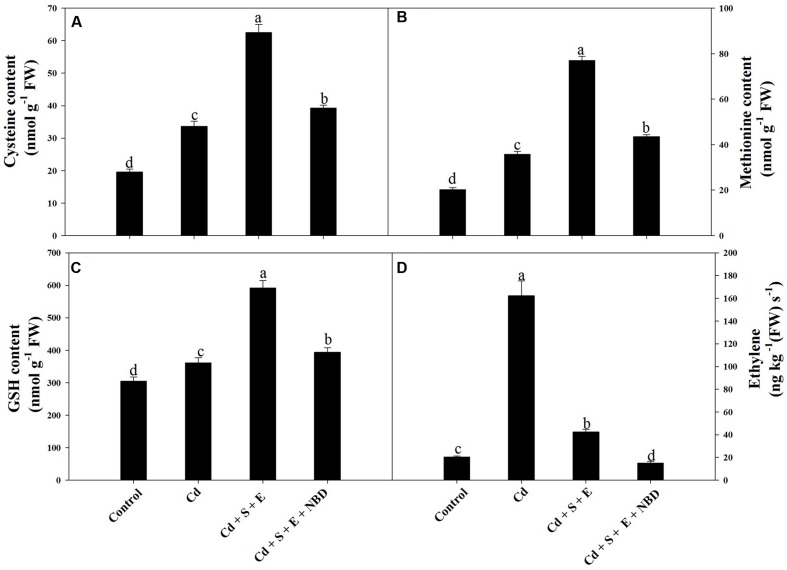
**Content of Cys **(A)**, Met **(B)**, GSH **(C)**, and ethylene **(D)** in mustard (*Brassica juncea* L.) at 30 DAS.** Plants were grown individually with 0 or 50 μM Cd (Cd), or with combined treatment of Cd + 1 mM SO_4_^2-^ (S) + 200 μL L^-1^ ethephon (E) in absence or presence of NBD. Data are presented as treatments mean ± SE (*n* = 4). Data followed by same letter are not significantly different by LSD test at *P* < 0.05.

Application of Cd increased content of Cys, Met, and GSH compared to control. Plants receiving S and ethephon in the presence of Cd increased content of Cys, Met, and GSH compared to Cd-stressed plant. Application of NBD with S and ethephon in the presence of Cd reduced Cys, Met, and GSH compared to Cd + S + ethephon treated plants. Plants receiving S and ethephon optimized ethylene in the presence of Cd and resulted in ethylene production higher than control but lesser than Cd-treated plants. This action of S and ethephon in Cd-treated plants on ethylene production was reversed with the application of NBD (**Figures 6A-D**).

**FIGURE 7:**
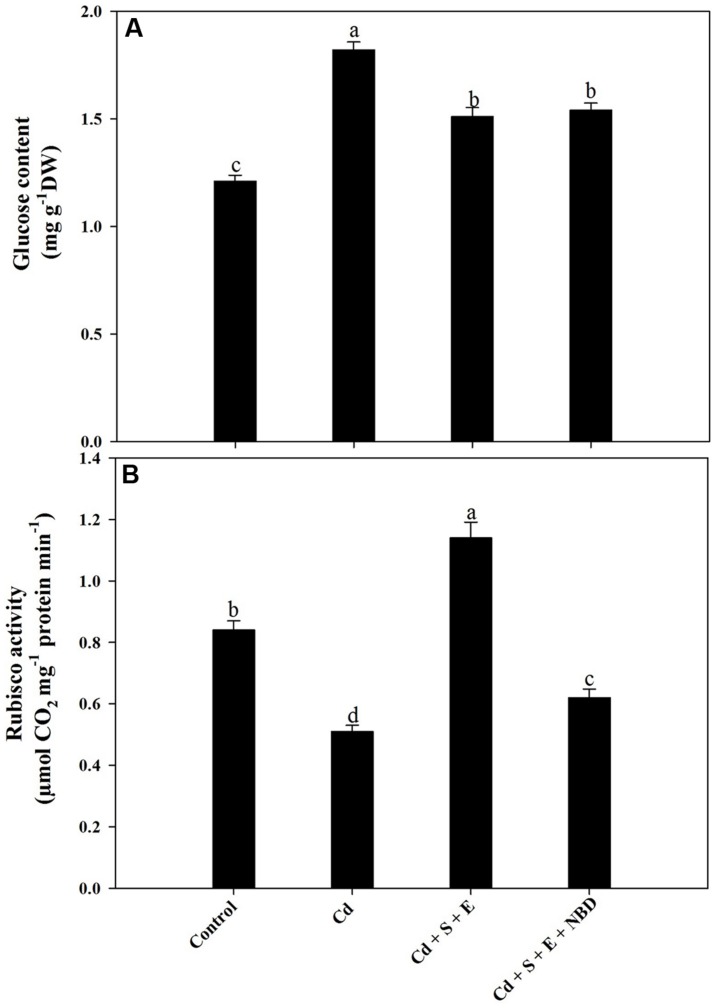
**Glucose content **(A)** and Rubisco activity **(B)** in mustard (*Brassica juncea* L.) at 30 DAS.** Plants were grown individually with 0 or 50 μM Cd (Cd), or with combined treatment of Cd + 1 mM SO_4_^2-^ (S) + 200 μL L^-1^ ethephon (E) in absence or presence of NBD. Data are presented as treatments mean ± SE (*n* = 4). Data followed by same letter are not significantly different by LSD test at *P* < 0.05.

Cadmium grown plants showed inhibition of Rubisco activity, however glucose content increased. Plants receiving S plus ethephon in the presence of Cd increased Rubisco activity compared to control and Cd-treated plants. Supplementation of NBD to S plus ethephon treatment in Cd-stressed plants reversed the effects and decreased Rubisco activity compared to control, but the Rubisco activity was higher than Cd-treated plants. In contrast, plants receiving S and ethephon in the presence of Cd increased glucose content compared to control but the content was lesser than Cd-treated plants. Application of NBD with combined application of S and ethephon to Cd-treated plants increased glucose content compared to control (**Figures 7A,B**).

**FIGURE 8:**
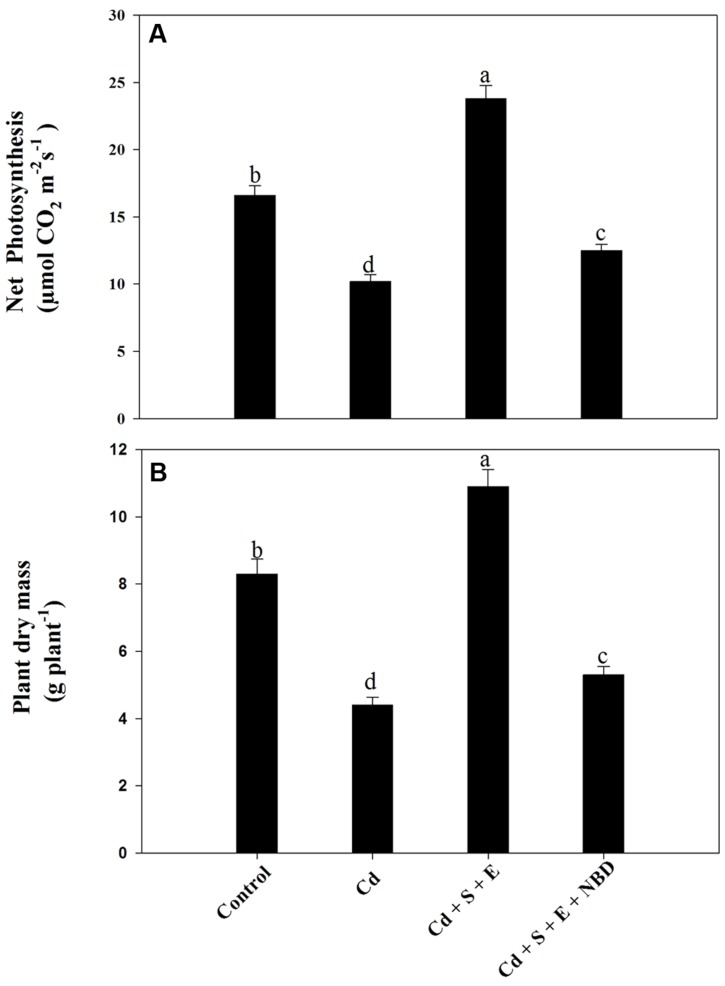
**Net photosynthesis **(A)** and plant dry mass **(B)** in mustard (*Brassica juncea* L.) at 30 DAS.** Plants were grown individually with 0 or 50 μM Cd (Cd), or with combined treatment of Cd + 1 mM SO_4_^2-^ (S) + 200 μL L^-1^ ethephon (E) in absence or presence of NBD. Data are presented as treatments mean ± SE (*n* = 4). Data followed by same letter are not significantly different by LSD test at *P* < 0.05.

Plants grown with Cd showed decreased net photosynthesis and plant dry mass compared to control. Application of S and ethephon together in the presence of Cd increased photosynthesis and dry mass compared to control and Cd-treated plants. However, NBD treatment reversed the effects of S plus ethephon. Photosynthesis and dry mass in this treatment (S + ethephon + NBD) were found higher than Cd-treated plants but lesser than control plants. Plant receiving combined application of S and ethephon in the presence of Cd increased photosynthesis and dry mass compared to control. However, NBD application in plants grown with S and ethephon in the presence of Cd decreased leaf area and plant dry mass (**Figures 8A,B**).

## Discussion

### Ethylene Application with S Decreases Cd Accumulation

Application of ethephon plus S maximally reduced Cd content in root and leaf. It has been shown earlier that S reduced Cd-induced oxidative stress in *Oryza sativa* (Hassan et al., 2005) and *B. campestris* (Anjum et al., 2008). Ethylene and S interacted to reduce oxidative stress induced by Cd in *B. juncea* and *T. aestivum* (Masood et al., 2012b; Khan et al., 2015b).

### Ethylene or/and S Reduces Cd-induced Oxidative Stress

Sulfur application and its assimilation potentially protect plants against stress. In this study, S and ethephon have similar effects on the alleviation of Cd stress by restricting oxidative stress. Application of ethephon and S in the presence of Cd disrupted Cd uptake and accumulation. The demand for increased thiols for removal of excess ROS under Cd stress was met with higher S-assimilation through increased activity of ATP-S and SAT consequently resulting in efficient detoxification of ROS. Application of S or ethylene minimized H_2_O_2_ and TBARS content, but the decrease was more prominent when ethylene and S were applied together to Cd grown plants (**Table 1**) apparently because of enhanced S-assimilation capacity of plants. This finding is in agreement with the earlier studies in which S reduced oxidative stress in *B. campestris* (Anjum et al., 2008), *Triticum aestivum* (Gaafar et al., 2012), *H. vulgare* (Astolfi et al., 2012), and *A. thaliana* (Bashir et al., 2013) under Cd stress. Role of ethylene has been found to reduce oxidative stress in *B. juncea* (Masood et al., 2012b; Asgher et al., 2014; Khan and Khan, 2014).

### Ethylene or/and S Reduces ROS Accumulation under Cd Stress

The combined treatment of ethephon and S resulted in the least staining due to the increased S-assimilation and GSH synthesis (**Figure 2**). The study provides evidence that ethephon and S influenced the accumulation of ROS in the plants. These findings demonstrated that the GSH synthesis was up-regulated when ethephon and S were applied to Cd treated plants, which in turn induced a strong ROS scavenging capacity and decreased production rate of 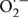. This is the first report regarding the response obtained from the ethephon with S in the inhibition of ROS accumulation using histochemical staining method.

### Involvement of Ethylene in S-induced Increase in Redox State (GSH/GSSG) under Cd Stress

Ethephon or/and S increased activity of ATP-S, SAT, and S content which resulted in increased content of Cys, Met, GSH, and redox state (**Table 1**; **Figures 3A,D**). Reports on the effect of ethephon and S on thiol (Cys and GSH) compounds under Cd stress are scanty. In this study, the increase in photosynthesis of Cd treated plants following ethephon and S application was coupled with increased thiol compounds. This suggests that ethephon and S induce tolerance to Cd stress and alleviate photosynthetic inhibition through ethylene by retaining high thiols. Increased activity of GSH potentially keeps redox environment for cell and protects plants against Cd toxicity by maintaining a high level of GSH content which is required for the regeneration of AsA (ascorbate) for the proper functioning of AsA-GSH cycle (Anjum et al., 2008; Asgher et al., 2014). However, reports on how ethylene and S influence Cys and Met synthesis under Cd stress are not available. Lancilli et al. (2014) have reported that S requirement to sustain thiol biosynthesis activated sulfate uptake in *B. juncea*. It is remarkable that high ethylene level in ethylene-sensitive cultivar signifies greater responsiveness to S-metabolism and ethylene synthesis and greater ethylene-induced physiological responses. It has been suggested that thiol metabolism increased in *H. vulgare* plants under Cd stress (Astolfi et al., 2012). It has been reported that GSH strongly reduced the Cd bioavailability by inhibiting its transports from root to shoot in *B. napus* and GSSG was not efficient in reducing Cd availability due to the lack of free thiol groups (Nakamura et al., 2013). Regulation of GSH by ethylene has been suggested under ozone stress in *A. thaliana* (Yoshida et al., 2009), under Cd, Ni, and Zn stress in *B. juncea*, *T. aestivum* (Masood et al., 2012b; Khan and Khan, 2014; Khan et al., 2015b), salt stress in *Vigna radiata. B. juncea* (Khan M.I. et al., 2014; Nazar et al., 2014).

### Application of Ethylene or/and S Reduces Glucose Content under Cd Stress

Ethephon and S reduced glucose-mediated photosynthetic repression of Cd grown plants. It has been reported that Rubisco expression was strongly inhibited by high endogenous glucose level in the ethylene-insensitive genotypes (Tholen et al., 2007). Masood et al. (2012a) have shown that glucose sensitivity increased under Cd stress that resulted in the inhibition of Rubisco activity and photosynthesis in *B. juncea*. The present work reports that in addition to the involvement of S-mediated ethylene production in alleviation of Cd stress through thiols synthesis, there also exists a relationship between ethylene and glucose and Cd tolerance. Iqbal et al. (2011) reported that ethephon application at each level of N increased ethylene and decreased glucose sensitivity, which increased photosynthesis and growth. Ethylene-sensitive cultivar has ability to overcome the inhibitory effect of glucose on photosynthesis and growth and was not sensitive to glucose developmental arrest. Reduction in Rubisco content and photosynthetic capacity in ethylene-insensitive *Nicotiana tabacum* genotypes was due to the absence of functional ethylene receptors (Tholen et al., 2008).

### Reversal of Stress Ethylene Production by Ethylene and S under Cd Stress

Ethephon or S treatment decreased ethylene production as compared to Cd stressed plants (**Figure 5B**) and resulted in favorable response with improvement in photosynthesis and growth. Plants grown with Cd were less sensitive to ethylene and exhibited adverse effects on photosynthesis and growth, but S or ethephon treatment increased the sensitivity of plants to ethylene. It has been reported by Iqbal et al. (2012a) that ACS activity and ethylene production increased with the application of 200 μL L^-1^ ethephon compared to control. Application of ethylene and S optimizes ethylene production under Cd stress resulting in improved photosynthesis and growth. However, in the presence of Cd the requirement of S by plants increased for GSH synthesis, and resulted in reduced ethylene synthesis, which was adequate for favorable effect on photosynthesis and growth. The inhibition of Cd-induced stress ethylene by S or ethephon may result increase in sensitivity of plants to ethylene and promote thiol metabolism.

### Involvement of Ethylene in S-mediated Alleviation of Cd stress on Photosynthetic Characteristics

As S-assimilation leads to ethylene formation via Cys, it is likely that S influences ethylene sensitivity, and ethylene is involved in the control of thiols synthesis and alleviation of Cd stress. Cadmium stress produces stress ethylene which was minimized to optimal range with S and ethephon favoring thiols synthesis via S-assimilation pathway and protected photosynthetic apparatus from Cd-induced oxidative stress resulting in increased photosynthesis. The increase in photosynthesis with ethephon and S involved stomatal and non-stomatal limitations as the treatments increased stomatal conductance allowing more exchange of intercellular CO_2_ concentration on one hand and increased Rubisco activity and expression on the other. The combined effect of ethephon plus S was found more promising in increasing gas exchange parameters, chlorophyll content and Rubisco activity (**Table 2**). Earlier, it has been shown that ethylene increases photosynthesis by increasing stomatal conductance and Rubisco protein of *B. juncea* (Iqbal et al., 2011). The modulation of photosynthesis by ethylene may result from increased diffusion rate of CO_2_ from the atmosphere to the intercellular spaces through increased stomatal aperture (Acharya and Assmann, 2009). Tholen et al. (2007) found that ethylene insensitivity in *N. tabacum* resulted in decreased photosynthetic rate associated with a strong reduction of Rubisco protein content. The interactive effect of ethephon and S on the photosynthetic response in plants under Cd stress has not been worked out in detail. The reason for the increase in photosynthesis with ethephon and S was due to S-induced ethylene and ethylene-mediated changes in allocation of S to Rubisco protein and also through increase in stomatal conductance under Cd stress.

Reversal of photo-inhibition and damage to photosynthetic attributes caused by Cd was modulated by the application of S and ethephon equally (**Table 2**). The increase in efficiency of PS II by ethephon and S treatment under Cd stress involved increased electron transport that helped plants to limit singlet oxygen production resulting in increased PS II activity under stress. Recently, it has been shown that exogenously sourced ethephon significantly reversed metals-induced photosynthetic inhibition by modulating PS II in *B. juncea* by lowering metal-induced stress ethylene to optimal range ethylene (Khan and Khan, 2014). However, no report is available on the role of ethylene in S-mediated protection of photosynthesis involving PS II activity and thiols metabolism under Cd stress.

### Ethylene or/and S promote Growth Characteristics under Cd Stress

The increase in growth with ethephon and S is attributed to S-induced ethylene-mediated changes in photosynthesis. The maximum alleviation of Cd-induced inhibition in growth was observed with the combined treatment of ethylene plus S apparently because of more efficient production of thiols that resulted in maximum protection of photosynthetic apparatus and thus leaf area and plant dry mass in Cd-treated plants. It has been reported by Zhang et al. (2014) that ethylene was involved in alleviating Cd-toxicity in *N. tabacum*.

### Inhibiting Ethylene Action Reversed S-mediated Alleviation of Cd Stress

In the presence of NBD, ethylene formation was reduced due to inhibition of ethylene action as it is a competitive inhibitor of ethylene, and by binding to ethylene receptors it reduced ethylene formation through autocatalytic regulation. Thus, ethylene concentration determines sensitivity and photosynthetic and growth responses. The action of both endogenous and exogenous ethylene can be modulated by the use of NBD and ethephon together. A critical threshold of ethylene concentration with sensitivity determines the overall response of plants. It may be said that ethylene-sensitive cultivar used in the present study had a lower threshold value for the response, and ethylene synthesis after ethephon application in Cd-stressed plants lowered stress ethylene to optimal level for maximum response.

Further, the content of glucose increased with Cd alone but the glucose content was reduced with S under Cd stress where the ethylene evolved was perceived by the plants and thus promoted photosynthesis by reducing the negative effect of glucose on Rubisco. Earlier, it has been reported that Rubisco expression was strongly inhibited by high endogenous glucose level in ethylene-insensitive plants (Tholen et al., 2007). In the present study, increased ethylene sensitivity in mustard plants metabolized increased glucose content that had negative consequences for Rubisco and photosynthetic capacity in Cd-treated plants. Ethylene induced in plants treated with Cd was responsible for photosynthetic inhibition, but NBD given with ethephon and S to Cd-treated plants showed lesser reduction in net photosynthesis and dry mass compared to Cd treatment alone (**Figure 8**). It appears that receptors were not available for stress ethylene induced by Cd as all the receptors were occupied by NBD resulting in lesser adverse effects of Cd-induced ethylene. Study on *B. juncea* grown with Ni and Zn stress by Khan and Khan (2014) showed that ethylene induced in plants treated with Ni and Zn stress was responsible for photosynthetic inhibition, but NBD treatment resulted in lesser reduction in photosynthetic attributes compared with Ni and Zn treatment alone.

## Conclusion

It may be concluded that the adverse effect of Cd on photosynthesis and growth was reversed more conspicuously when ethephon and S were applied together by reduced Cd-induced ROS production through optimization of ethylene level, increased S-assimilation and thiol (Cys, GSH) production. Moreover, this combined treatment reduced glucose mediated photosynthetic inhibition under Cd stress. The use of ethylene action inhibitor, NBD also confirmed that ethylene was responsible for S-induced alleviation of Cd stress. The present work suggests that S supplementation in agricultural system and ethylene optimization may be adopted for augmenting photosynthesis and growth under Cd stress.

## Author Contributions

NK conceived the concept and MA performed the experiments. TP, AM, MF, and MK helped in plant sampling and data analyses. NK and MA wrote the manuscript. All authors read and approved the manuscript.

## Conflict of Interest Statement

The authors declare that the research was conducted in the absence of any commercial or financial relationships that could be construed as a potential conflict of interest.
